# Dying online: live broadcasts of Chinese emerging adult suicides and crisis response behaviors

**DOI:** 10.1186/s12889-016-3415-0

**Published:** 2016-08-11

**Authors:** Jing Ma, Wei Zhang, Keith Harris, Qiang Chen, Xiaolin Xu

**Affiliations:** 1 College of Public Administration, Huazhong University of Science and Technology, Wuhan, Hubei 430074 People’s Republic of China; 2 School of Medicine and Health Mangement, Wuhan, Hubei 430030 People’s Republic of China; 3 School of Medicine, University of Tasmania, Hobart, TAS Australia; 4 School of Psychology, University of Queensland, St Lucia, QLD Australia

**Keywords:** Live-blogcast suicide, Social media, Suicide prevention, Online behaviors, Suicidal communications, Cyberbully, Crisis response

## Abstract

**Background:**

Social media and online environments are becoming increasingly popular and integral to modern lives. The online presentation of suicidal behaviors is an example of the importance of communication technologies, and the need for professionals to respond to a changing world. These types of behaviors, however, have rarely been scientifically analyzed. This study aimed to examine the behaviors of both suicide broadcasters and their audience, with attention on prevention/crisis opportunities.

**Methods:**

Multiple case studies were employed to explore live-broadcast suicide by Chinese emerging adults (aged 18-25 years). Six cases were selected (four males, two females; aged 19-24, *M* = 21.60, *SD* = 2.25), retrieved from 190 public documents (case range = 5 to 32; *M* = 11.50, *SD* = 10.37). A qualitative study based on grounded theory was adopted. Information on case background, stages, participants and their behaviors were collected.

**Results:**

(1) Five stages of blogcast suicide incidents were revealed, including: Signaling, Initial reactions, Live blogcast of suicide attempts, Crisis responses, and Final outcomes. (2) Common behavioral trends (*e.g.,* comforting, verbal abuse) were identified from the blogcast participants (*e.g.,* active audience, peers, parents and police). (3) Suicide blogcasters exhibited tendencies to communicated signs of pain and cries for help.

**Conclusions:**

This multi-case study found live presentations of suicidal behaviors offered unique opportunities to respond to suicidal crises, and also to learn more about the relationships between suicidal people and potential help sources. Findings showed many audience members wanted to be helpful but lacked appropriate skills or knowledge. Others engaged in suicide cyberbullying. The social media is an environment in the making. This study revealed that increasing knowledge and skills for crisis response and suicide prevention is needed. Such efforts could lead to empowered netizens and a more hospitable online world.

## Background

Social media has become an integral part of many people’s daily lives. The number of people using social media, has surpassed 208 million, accounting for 29 % of the global population [1]. The online environment enables self-expression, instant interaction, and communications with people unknown offline [2]. Social media can enrich the content and scope of personal communications and also facilitate the self-presentation of undesirable behaviors [3, 4]. According to the WHO, suicide is the second leading cause of youth death globally [5]. As more young suicidal people are going online, there may be increased opportunities for help, prevention and crisis response.

Emerging adults, defined here as those aged 18–25 years, are of particular interest because of their unique attributes regarding online behaviors and mental health factors [6–8]. Their online suicidal behaviors are no exception [9–12]. As for the connection between emerging adult suicide cases and social media development, some scholars have highlighted the potential negative impact upon people exposed to online information encouraging suicide [13]. Netizens may intentionally or otherwise increase suicidal feelings and behaviors in others, or alternatively, play a positive role by assisting people at risk for suicide through access to rescue or support [14–16]. Studies have suggested that social media-based suicidal communications can be an unorthodox way of help-seeking [11]. Social media can be used for mental health support or online mental service stations. Many organizations and individuals, such as social services and community professionals, volunteers or non-profit organizations, provide suicide prevention mental health services, assessment, psychotherapy and counseling [17, 18].

With respect to specific crisis responses and prevention strategies, researchers have highlighted the important roles played by parents [19, 20], educators [21, 22], peers [23, 24] and community members in providing assistance to suicidal individuals in an offline environment [25, 26]. Matlin et al. revealed that family and peer support helped reduce youth suicide rates, and peer support and community connectedness moderated depressive symptoms and suicidality [27]. However, with the exception of online support groups, prevention strategies in social media environments have not been well explored [9]. Few studies have examined the roles of participants involved in preventive strategies or crisis responses [28], with even less attention paid to those in non-Western societies [14]. For China, despite the tremendous development of social media, little is known about its impact on suicidal people its potential for prevention/crisis response.

### Chinese social media, weibo

Weibo, a major social media platform in China, includes weblogs enriched with features for social networking [29]. Weibo enables users to generate their own content, including text, images, audio and live-streaming video. Each Weibo user has a unique username, and others can search, view and connect with them by searching content or username. Weibo data was open-accessed, similar to Twitter, where anyone can check another’s profile. It is estimated that, in the first half of 2011 alone, Weibo users surged to 195 million from an initial 63 million, and the majority were youth [30]. As a free-speech platform, Weibo offers Chinese an unprecedented opportunity for sharing information and expressing opinions and emotions [14]. However, undesirable information and behaviors have also occurred on Weibo. At the end of August 2010, the first live-broadcast Weibo suicide case (blogcast suicide) was reported. Since then, quite a number of similar cases have been reported [31], which have not been scientifically studied.

### Suicidal communication theories

Although various theories are related to suicide and suicidal factors, this study was interested in how suicidal people communicate their suicidality– which might be evidenced through their online behaviors. The online environment can be exploited as a space to express suicide-related emotions due to anonymity and privacy features [32]. When engaging in suicidal communications, people were more likely disclose their true self [33]. This can be a result of the online one-to-one or group disinhibition effect [34–36].

Farberow and Shneidman [37] theorized that all suicides are cries for help. Communication of suicidality is a behavior that can increase social support and protect a suicidal person from danger at their own hands [12, 38, 39]. Blogcast suicide could be a modern version of a cry for help, but there is little data proving that hypothesis. Shneidman [40] further hypothesized that people become suicidal due to unmet psychological needs, following on Murray’s [41] system of needs model. Beck and colleagues [42], however, proposed that suicidality stems from hopelessness, which could be evidenced through blogcast statements of hopelessness as an overriding feeling. Evidence supporting those theories would be found by suicide broadcasters making communications identifying human needs or emotions in their suicidal behaviors. With the exception of Wiggins and colleagues’ [16] study, previous research mostly focused on suicidal motivations with less attention paid to online suicide processes and crisis responses.

### Study aims

The suicidal communication process may help explain the suicidal individual’s emotional needs (*i.e.,* consolation, understanding or care) as presented on social media. Emerging adults are the main adopters and heavy users of social media, comprising over 40 % of Weibo users [43]. Based on media attention, they are the most common blogcast suicide group. This study, therefore, focused on the emerging adults blogcast suicide behaviors (including both suicide attempts and completed suicides), as well as the behaviors of the people who participated in those incidents – in a Chinese context. The research goals were three-fold: (a) to identify the process and stages of blogcast suicide incidents from Chinese emerging adults; (b) to examine the participants, their crisis responses and the impact they had during the suicide process; and (c) to determine possible means for preventing suicides through Weibo and other social media. To accomplish these goals, several cases of online suicides or attempts, by Chinese emerging adults were selected and examined through qualitative methods.

## Methods

### Case selection

This multiple case study targeted emerging adult, aged 18 to 25, live-broadcasted suicide incidents that occurred on Weibo. In line with case study methodology, various sources of data were collected to obtain a comprehensive picture of the suicide incident process on Weibo and participants’ behaviors.

To locate information sources covering target suicide incidents, we used Baidu, the most comprehensive search engine in China, with the following keywords “suicide incident”, “Weibo”, “emerging adult” and “live broadcast”. Numerous search combinations were attempted until all published cases were located. That strategy identified 11 cases. Each case was then researched separately, by username *etc.*, to obtain all possible information on the case and incident. Five cases were then eliminated due to failure to meet the age range and broadcast platform, or insufficient information (*i.e.,* the suicide blogcasters posted little suicide-related information; the posts had no appearance of being life-threatening or resulting in death; the suicide event was reported in only a single news story available for public consumption). In total, six cases and 190 news articles from 69 professional news agencies and available blog records of the blogcasters were obtained. Each case had 5 to 32 supporting articles (*M* = 11.50, *SD* = 10.37).

### Data collection

Data on the suicide incidents, stages/process, participants and their behaviors, was collected. The resulting data consisted of: (a) Background: information on the suicide incidents and blogcasters, including incident dates, online platform, age, sex, location, expressed reasons for suicide; (b) Stages: process of the suicide incidents, including duration, key factors of blogcast suicide and outcomes; (c) Participants and their behaviors, relation of the participants to the suicidal incidents and expressed opinions. These methods were pilot tested through two prior cases, which resulted in the final data extraction procedures.

### Case analysis and cross-case comparisons

Grounded theory for qualitative content analyses was followed [44, 45]. An open coding system was used with the initial dataset, followed by axial coding and selective coding. Cases were analyzed with NVivo v. 8 (QSR International).

Articles were examined by the study’s first two authors to identify and label content into appropriate codes. To generate the coding framework, the first author inductively analyzed the reports of the selected cases and classified them into an initial category. Two trained research assistants then provided their feedback to develop the final coding framework [46, 47]. The data were reviewed, organized and interpreted by the authors independently during the analysis, and alternative interpretations were continually discussed. Half of the cases were randomly selected and coded by two authors independently to assess inter-coder reliability [48]. The classification showed adequate inter-rater reliability, Kappa = .81. Next, discussions led to classification protocol for the remaining cases.

A cross-case analysis method was followed to examine similarities and differences between the cases. That method identified a tentative typology of stages and participants’ crisis response/prevention. Further comparisons culminated in a typology of participants behaviors.

### Blogcasting suicide cases

In the selected cases, ages ranged from 19 to 24 years (*M* = 21.60, *SD* = 2.25). Two of the six cases were female. All suicide incidents were blogcasted through the Sina Weibo, with one exception (the specific Weibo platform for Case 6 was unknown). The blogcast suicide lasted approximately 3 to 8.5 h (*M* = 7.00, *SD* = 6.22).

With respect to the precipitating incidents, over half (66.6 %) showed evidence of relationship break-ups and the remainder appeared to be related to family problems. Blogcasters in Cases 2 and 5 died by their own hands, while the other four were rescued. Details are presented in Table 1.

**Table 1:** Emerging adults live-blogcast suicide incidents in Chinese Weibo

Case	Duration	Location	Sex	Age	PE	Method	Result	Participants
1	5/16/2011	Shaanxi	Male	24	Family conflicts	Wrist-cutting	Rescued	Active audience, newspaper reporter, roommate, police
	14:28-23:00							
2	9/1/2011	Hubei	Male	24	Break-up (likely)	Overdose	Died	Active audience, colleagues and fans, family members
	01:07-19:23							
3	10/23/2011	Beijing	Female	22	Break-up	Overdose	Rescued	Active audience, police
	22:13-1:20							
4	3/7/2012	Sichuan	Female	22	Break-up	Wrist-cutting	Rescued	Active audience, friends and ex-colleagues, family members, police
	02:50-6:00							
5	11/30/2014	Sichuan	Male	19	Break-up	Charcoal-burning & Overdose	Died	Active audience, friends, family members, police
	07:48-14:00							
6	12/1/2014	Fujian	Male	19	Health and family issues	Overdose	Rescued	Active audience (celebrity), police
	17:00-21:30							

Note: The duration began from the time when the blogcaster posted suicide-related information and ended with his/her last relevant post, excluding those messages indicating negative mood in general. *PE* precipitating event

Based on the case analysis, the participants could include active audience (strangers on social media who viewed the suicidal incident and/or communications prior to the suicidal incident and also communicated with the suicide blogcaster), family members, friends, colleagues, fans and police. In addition, there would have been lurkers, who made no known communications with others but only watched the incidents. As there is no recorded data on the details of lurkers, they were not included in the following analyses. Though different in scope, these audience types are consistent with previous research [14, 19, 28]. We divided theses participants into four groups by their occupation and relationships with the blogcasters: active audience, peers (young acquaintances including friends, young family members, colleagues, schoolmates and fans who contacted the blogcaster at least once), parents and police.

## Results

### Processes of live-blogcast suicide

**Fig. 1:**
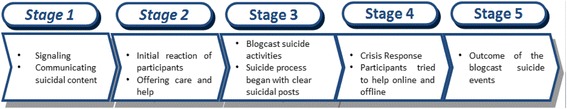
The stages of live blogcast suicide events

The cross-case analyses revealed five stages were included in live-blogcast suicide incidents. Figure 1 illustrates these five stages, from Signaling pre-suicide information to Final outcomes.

#### Stage 1: Signaling – pre-suicide information

Before blogcast suicide incidents, the blogcaster communicated with others online. Those communications were made several hours to a few days before the live suicide attempt. Most commonly, these messages showed evidence of negative self-perceptions or hopelessness about their future, such as “unhappy”, “lonely”, “painful” and “unlikeable”. In the selected cases, all the suicide blogcasters expressed this type of message varied from 1 to 60 posts (*M* = 13.00, *SD* = 21.11).

In Case 2, the suicide blogcaster, a well-known radio DJ, posted five messages referring to his hopelessness and suicidal intent, including *“My whole life is a joke. I have led an empty life for one month, but I could not find an answer … Let me leave this world quietly…I want a simple and happy life, but I get no chance”*.

The blogcaster in Case 5 posted negative messages one day before completing suicide, such as *“The body is useless when the heart has died”*. After this post, he sent another 44 Weibo messages over one and a half hours. In his later posts, he showed vulnerability due to perceptions of betrayal, and also frequent self-denial (*i.e.,* “*Living is so boring to me. Betrayed by my family, my friends and my lover, it meaningless for such an idiot as me to live in this world”*), and then mentioned cutting his wrists.

#### Stage 2: Initial reactions

When the blogcasters’ behaviors showed signs of serious suicide intent (*i.e., “I think wrist-cutting suits me the best”* in Case 5, and *“I feel relieved to leave the world”* in Case 2), the participants, including active audience members, some peers and family members, expressed concern and consoled the blogcasters.

In Case 2, after the blogcaster released negative messages on Weibo, his colleagues and fans immediately responded with caring comments. His brother also called their mother to find out what was wrong. In Case 5, the suicide blogcaster displayed some positive reactions to the emotional support shown by his audience. He posted *“Thanks for the positive energy from my ex and everyone else. I will have a good sleep and go out shopping tomorrow morning”*. The post mentioned social support from friends and some active audience members, which appeared to partly meet his psychological needs. Comments of “good sleep” and “shopping” may have indicated emotional peace.

#### Stage 3: Live blogcast of suicide attempts

For the six cases, the participants failed to prevent any of the suicide attempts. Then the suicide blogcasters began their suicide process, which included posting images and videos of potentially lethal acts (*N* = 5), and posting comments indicating suicidal intent (*N* = 6).

In Case 1, the blogcaster displayed his left wrist with a deep cut and the word “*Enough!*” typed in red at 14:28 on Weibo. Two hours later, he posted a second picture, showing the blood from his cut wrist gradually dripping on the floor, and posted “*Drop by drop, what a long time*”. Later, at approximately 20:00, a third picture was posted showing his wrist dripping with blood and his palm turned pale, with the text “*XXX* (someone’s name), *is that enough?*”

In Case 5, the blogcaster displayed almost every detail of his fatal suicidal behaviors through 38 posts. The posts illustrated his suicidal intent, his purchase of sleeping pills and charcoal, an earlier suicide attempt, a possible cry for help, and his final suicidal behaviors. “*Today is 30*^*th*^*November. It is still early now, and I am not sure if the hospitals and supermarkets are open yet. I thought for a long time calmly and decided to leave. Do not persuade me. Hope the last minute will not be too much pain*”.

#### Stage 4: Crisis responses

Once the actual incidents began, most peers, parents, police and other active audience members expressed concerns and tried to save the blogcasters. Peers and parents offered immediate information to the police and attempted to rescue the suicide blogcasters. Some active audience members called the police providing the blogcaster’s registration information and the content of their posts. In Cases 3 and 5, active audience informed the police through @police official Weibo.

In Case 1, when the blogcaster presented a second image of his cut wrists, active audience tracked his IP address and located him. A journalist from *Huashang Newspaper* involved in the live incident also helped [49]. The blogcaster’s roommate returned to help the moment he saw the posts. In Case 4, a user in another city found the incident at midnight. She tried to comfort the blogcaster and called the police and local friends for help. Through these online and offline efforts, the suicide was terminated. In Case 6, the blogcaster and Yongyuan Cui, a famous Chinese TV host, were mutual friends on Weibo. When the blogcaster posted messages of suicidal intent, Cui showed his concern and repeatedly sent him encouraging information via Weibo, such as *“Everything will be fine”,“I am not sure what bothers you, but that’s not your fault in any sense”*.

#### Stage 5: Final outcomes

This was the last stage of the live-blogcast suicide. In the six cases, four were rescued in Cases 1, 3, 4 and 6. In Case 1, the blogcaster was saved by his roommate. In Case 3, police rescued the blogcaster. In Case 4, the family came and saved the blogcaster. In Case 6, the blogcaster abandoned the attempt after receiving support from Cui and other active audience members. The blogcasters of Cases 2 and 5 died by their online suicide behaviors. In Case 2, the blogcaster took a lethal dose of pills early in the morning after updating his last status. In Case 5, although the police and family members were able to get the suicidal blogcaster to the hospital, he died from charcoal-burning and overdose.

### Various behaviors from participants

The participants made various attempts to prevent the live-blogcast suicides. Those behaviors may differ by the relationship between the participants and the blogcasters, or by other factors. Some participants even incited the suicidal behaviors. Table 2 summarizes preventive efforts by participants’ type.

**Table 2:** Classification of participants’ behaviors towards the original message

Behaviors	Participant type	Translated examples
Offering care and showing empathy	Active audience	Case 1: “I hope this stranger is just unable to get over only for a while. Please think about your family and your friends. How miserable they will be if you committed suicide”. “Suicide is an irresponsible way to yourself and the people who love you”.
	Peers	
Searching useful information, calling for information and help	Active audience	Case 1: “His IP and telephone show that he is in Yulin. Call the police now.”
	Peers, police	Case 4: “Anyone in Chengdu happens to know the suicide broadcaster? Call the police immediately!”; “The policeman called me again but I really don’t know the situation.”
Making cynical or indifferent comments, “like” and incitement	Active audience	Case 3: “She must do this (referring to the suicidal behaviors on Weibo) for fame”, “God knows?!”
		Case 5: “Why are you (referring to the suicide broadcaster) still alive?”
		Case 6: “This person deserves to die.”
Expressing shocked feelings	Active audience	Case 1: “Horrible! I could not fall asleep tonight.”
On-site rescue actions	Peers, Parents, Police	All the 6 cases: Rush to the suicidal location for rescue

In the selected cases, most participants played similar positive roles in crisis responses, although slightly different in scope and pathway. The active audience, unacquainted with the blogcaster in the offline world, represented the largest documented subgroup and had notable influence on blogcast suicide incidents. In contrast with active audience and peers’ quick respond and timely rescue, parents showed insufficient impact during the process. Some active audience members, however, played conflicting roles throughout the process, as illustrated below.

#### Consoling the suicide blogcasters

Most participants responded positively by showing immediate concern for the blogcasters. In all 6 cases, the suicide-related posts drew wide attention. In Case 5, the active audience persuasion worked for a time. In Case 6, the celebrity Cui played a key role in consoling the suicidal person.

#### Providing information for timely rescues

Once a potential suicide was identified, active audience and peers searched for information on addresses and phone numbers of the blogcasters. In Cases 1, 4, 5 and 6, the active audience employed *cyber manhunts*, a collaborative search by ordinary netizens to obtain accurate information on a specific person, which was invaluable in rescue. In Case 4, one active audience member made long-distance calls to report the incident and motivated others to locate the blogcaster. Eventually, through several layers of relationships, she contacted the blogcaster’s friends and family.

#### Reporting to the police & rescue

When active audience and peers had been aware of the seriousness of the blogcast suicide incidents, they sent the information to the police department. Next, police, parents and peers became involved in on-site rescues, such as the rescue in Cases 1, 3 and 4. However, due to insufficient information, they were sometimes too late (*i.e.*, Cases 2 and 5).

#### Cynical and indifferent attitudes

Some active audience members suspected the blogcaster’s motivations by openly analyzing and reporting on their identity and previous Weibo content. They claimed the blogcaster was performing a live suicide show for public attention (*i.e.*, one active audience member criticized the suicidal model in Case 3, posting *“I think she did this for fame”*), which was more common for the public figure cases (the DJ in Case 2 and the model in Case 3). Those active audiences focused on their own perceptions of the lethality of the suicidal behaviors, rather than on the blogcasters’ feelings and personal situations.

#### “Likes” and incitement

In Case 5, quite a few members “liked” the suicidal messages and incited the blogcaster to suicide (*i.e.*, *“I do not care whether this person die or not” in Case 5*). In Case 6, after the blogcaster was discovered to have abused an older beggar years ago, many users were enraged and intensively criticized him (*i.e., “You deserve to die”* posted by one active audience member in Case 6).

The cynical and indifferent comments from active audience could be recognized as cyberbullying from a different perspective. In Case 5, the blogcaster responded to the cruel comments as, *“You are happy now. Sorry, everyone. I will leave the rest to God”*, possibly denoting his helplessness. In Case 6, the rescued blogcaster said that he did not know how to respond to those who urged him to kill himself online.

## Discussion

Online suicide attempts and completions appear to be a growing phenomenon. This study provided detailed analyses on the behaviors of Chinese emerging adult suicide blogcasters and participants. Findings revealed important information can be obtained from accounts of these incidents, which could contribute to a better understanding of suicidal people, those who encourage suicidal behaviors in others, and perhaps most importantly – how suicide could be prevented in an online/wired environment. From this multiple case study, a significant proportion of the participants’ behaviors appeared to be positive, characterizing by showing care and taking actions to help the suicidal blogcasters. The surge in live blogcast suicide incidents presented the individual’s self-perceptions– feelings of unhappiness, insecurity, loneliness, and poor self-image. Those online struggles of the suicidal mind, between their wish to live and their wish to die, are not often recorded in such a way to provide valuable support and prevention information.

### Blogcasting suicide: continuous cries for help

For the cases of this study, most of the emerging adult suicide blogcasters appeared to gain the attention of a particular person (*i.e.*, the ex-girlfriend or ex-boyfriend), perhaps hoping for a reunion. Their suicidal communications, the cry of pain and for help [37], was often answered through the caring responses of some participants, even if they were not the intended message recipients. Similar to recent work [16], opportunities for providing social support and crisis intervention were identified.

Beck and colleagues [50, 51], through the Suicide Intent Scale, demonstrated that individuals who make active precautions against discovery of their suicide attempt are at significantly higher risk of dying by suicide than other suicidal individuals. Clearly, blogcasting suicide is antithetical to hiding suicidal intentions. Evidence was found that suicide blogcasters received positive feedback and social support which are important suicide protective factors [12, 32, 38, 39]. Cries for help resulted in positive displays of support, evidenced through numerous statements made by close associates and online participants. However, that does not fully preclude the possibility of fatal attempts, or the sincere emotional psychache the actors are experiencing, as demonstrated in the current study. The ambivalent nature of the suicidal mind presented by the blogcasters uncovered their struggle between life and death. In conflicting situations, some blogcasters showed desires to connect with others and to heal their internal wounds. Those connections can offer realistic, but challenging, opportunities for suicide prevention.

In addition, there were also important cultural factors at play. Within the Chinese context, people, including emerging adults, who experience mental health problems are unlikely to openly communicate mental health issues offline or see mental health professionals [52]. With the advance of Information Communication Technology (ICT), some people prefer to turn to the social media for help or release [11, 53].

### Impact of responses: weak ties and peers effects

Participants’ responses in the blogcast suicide incidents may contribute to a beneficial environment in helping blogcasters to recover from suicidal shadow. Timely responses from peers could be life-saving, and so do other active audience members.

#### Weak ties effects

The loosely tied interpersonal relationships between blogcasters and most active audience are *Weak ties,* which might be more helpful than a closed group in terms of responding to live suicide incidents [14, 54]. On social media, weak ties link a person to a friend who has one or more friends who are not friends with the individual [55]. Our study found that active audiences’ activities contributed to social support and rescue information, providing some evidence that such distant connections can yield positive results.

#### Immediate peer response

From the case analyses, we found that compared to parents, peers were more likely to observe the suicidal messages sent by the blogcasters, and offered consolation and rescue actions. Possible illustration is that peers always have similar social media habits and general shared interests, which implied opportunities in emergency responses.

#### Risks of comments

Previous research has demonstrated that the experience of cyberbullying may result in depression among youth and increased suicide risk [56, 57]. Our research further found that cynical and indifferent comments from some active audience members may increase likelihood of suicide. This cyberbullying may have negative effects on the emotions of the blogger and on other individuals who are exposed to the information [14].

### Improving online suicide prevention

Emerging adults should have more opportunities to notice when their peers are at risk of suicide. However, our study revealed little evidence that emerging adults could adequately discern suicide intentions and take appropriate measures to respond effectively. That may due to insufficient suicide awareness and knowledge for detection and interventions of suicidality. In China, professional mental health services and training on suicide prevention are not widely accessible [58].

This study revealed sound potential for online suicide prevention. Active audience members displayed compassion and a willingness to take positive actions, although they appeared to lack sufficient knowledge on how best to respond. Considering the effectiveness of suicide prevention training and the low-cost of social media, social media should be utilized more for suicide prevention training. More best-practice materials could be made available in more languages, and better distributed to social media platforms, not only to professional organizations. Within the social media environment, that information can be widely accessed through various platforms, such as Weibo, search engine sites, and other easily accessible sites. Additionally, social media could also be a good platform for the active audience with professional skills to offer online support. Emergency services should institute clear protocols for collecting information and responding to life-threatening crises.

### Study limitations and future directions

This study’s analyses included six live-blogcast suicide cases focusing on emerging adults, and the data was dependent on online news articles. It is possible that some information was not accurate, and there was no data for other age-groups. The information was incomplete as news services do not include all information due to ethics and space limitations [59, 60]. In addition, the full communications by the suicide blogcasters and the participants were not available. For each individual case, their history of suicidal affect, cognition, behaviors, and psychiatric background were not included in their blogcasts. Such data, indicating the variation in the depth of suicidality (*i.e.*, the degree of suicidal intent), is necessary to better understand the relevant internal factors of the blogcasters. Discourse analysis or psychological autopsies could provide further qualitative information on these incidents [16, 61–63]. Online suicide incidents may also differ by online platforms, as the user environment will affect communications and other factors.

## Conclusions

This study provided multiple case analyses of Chinese emerging adult live-blogcast suicide. Five stages of online suicide incidents were identified: Signaling, Initial reactions, Live suicide attempts, Crisis responses, and Final outcomes. We also analyzed various players in this online life-death drama: active audience (who were unacquainted with the suicidal individual offline, but participated in the incident), peers, parents, and police. Many participants provided help for the suicidal blogcaster, including consoling, reporting information to emergency services and timely rescues. There were also less desirable factors of these online incidents, such as suicide cyberbullying and a lack of suicide prevention awareness or training of most participants. Our study suggests that online suicide behaviors can be prevented if there is greater awareness of suicidal symptoms and useful protocols to follow when encountering suicidal communications.

## Abbreviations

ICT, Information Communication Technology; WHO, World Health Organization
